# *In Vitro* Assays Using Primary Embryonic Mouse Lymphatic Endothelial Cells Uncover Key Roles for FGFR1 Signalling in Lymphangiogenesis

**DOI:** 10.1371/journal.pone.0040497

**Published:** 2012-07-06

**Authors:** Jan Kazenwadel, Genevieve A. Secker, Kelly L. Betterman, Natasha L. Harvey

**Affiliations:** 1 Division of Haematology, Centre for Cancer Biology, SA Pathology, Adelaide, Australia; 2 School of Medicine, University of Adelaide, Adelaide, Australia; Feinberg Cardiovascular Research Institute, Northwestern University, United States of America

## Abstract

Despite the importance of blood vessels and lymphatic vessels during development and disease, the signalling pathways underpinning vessel construction remain poorly characterised. Primary mouse endothelial cells have traditionally proven difficult to culture and as a consequence, few assays have been developed to dissect gene function and signal transduction pathways in these cells *ex vivo*. Having established methodology for the purification, short-term culture and transfection of primary blood (BEC) and lymphatic (LEC) vascular endothelial cells isolated from embryonic mouse skin, we sought to optimise robust assays able to measure embryonic LEC proliferation, migration and three-dimensional tube forming ability *in vitro*. In the course of developing these assays using the pro-lymphangiogenic growth factors FGF2 and VEGF-C, we identified previously unrecognised roles for FGFR1 signalling in lymphangiogenesis. The small molecule FGF receptor tyrosine kinase inhibitor SU5402, but not inhibitors of VEGFR-2 (SU5416) or VEGFR-3 (MAZ51), inhibited FGF2 mediated LEC proliferation, demonstrating that FGF2 promotes proliferation directly via FGF receptors and independently of VEGF receptors in primary embryonic LEC. Further investigation revealed that FGFR1 was by far the predominant FGF receptor expressed by primary embryonic LEC and correspondingly, siRNA-mediated FGFR1 knockdown abrogated FGF2 mediated LEC proliferation. While FGF2 potently promoted LEC proliferation and migration, three dimensional tube formation assays revealed that VEGF-C primarily promoted LEC sprouting and elongation, illustrating that FGF2 and VEGF-C play distinct, cooperative roles in lymphatic vascular morphogenesis. These assays therefore provide useful tools able to dissect gene function in cellular events important for lymphangiogenesis and implicate FGFR1 as a key player in developmental lymphangiogenesis *in vivo*.

## Introduction

The cellular processes underpinning the growth and development of lymphatic vessels (lymphangiogenesis) include proliferation, migration, adhesion and lumen formation. All of these events need to be precisely orchestrated in order to build a lymphatic vascular network able to function optimally to maintain tissue fluid homeostasis, coordinate immune cell trafficking and absorb lipids from the digestive tract. Aberrant lymphangiogenesis is associated with a spectrum of human disorders including vascular malformations, lymphoedema, inflammatory diseases and cancer [1], [2]. Deciphering the genes and signalling pathways that control lymphangiogenesis is crucial in order to uncover targets to which new therapeutics able to combat these diseases can be designed.

While the study of lymphangiogenesis *in vivo* using animal models including the mouse, frog and fish has yielded invaluable information regarding the genetic pathways important for lymphatic vascular development [1], [2], the dearth of established assays to manipulate primary lymphatic endothelial cells (LEC) isolated from mouse tissue in culture has restricted our ability to dissect lymphangiogenic signalling pathways *ex vivo*. Models established to study lymphangiogenesis *in vitro* include the culture of LEC isolated from bovine [3], [4], canine [5], human [5], rat [6] and ovine [7] collecting mesenteric lymphatic vessels or thoracic duct, and the culture of LEC isolated from adult mouse [8] or human skin [9]. Approaches to yield greater numbers of cells for analysis have included the culture of immortalised LEC from human [10], [11] or mouse lymphangiomas [12], [13], the introduction of telomerase to human LEC [14] and the isolation of SV40 large T antigen immortalised LEC from the thoracic duct of rats [15] and from various organs of mice [16], [17]. Conditions that promote the differentiation of LEC from embryonic stem cells have also been established [18], [19], [20]. Three dimensional models employed to study sprouting lymphangiogenesis *in vitro* include the culture of small segments of rat [21] or mouse [22] thoracic duct in collagen gels, culture of human LEC subjected to flow in collagen [23] or fibrin gels [24] and the culture of beads coated with human LEC or LEC spheroids in fibrin gels [25]. Each of these approaches has limitations; very small numbers of primary cells can be purified from collecting lymphatic vessels, there is considerable heterogeneity in the endothelial cells that line large collecting vessels compared to lymphatic capillaries, and immortalisation and extended culture alters the molecular properties and identity of LEC. Moreover, while the culture of primary human LEC over several passages has been feasible, the culture of primary mouse LEC has been challenging. We sought to optimise methodology for the isolation and short-term culture of primary embryonic mouse LEC in order to establish lymphangiogenesis assays able to: 1. Measure LEC proliferation, migration and tube formation in response to established and candidate pro- and anti-lymphangiogenic stimuli. 2. Assess gene function in defined aspects of lymphangiogenesis by utilising siRNA to silence gene expression in wild-type LEC.

**Figure 1:**
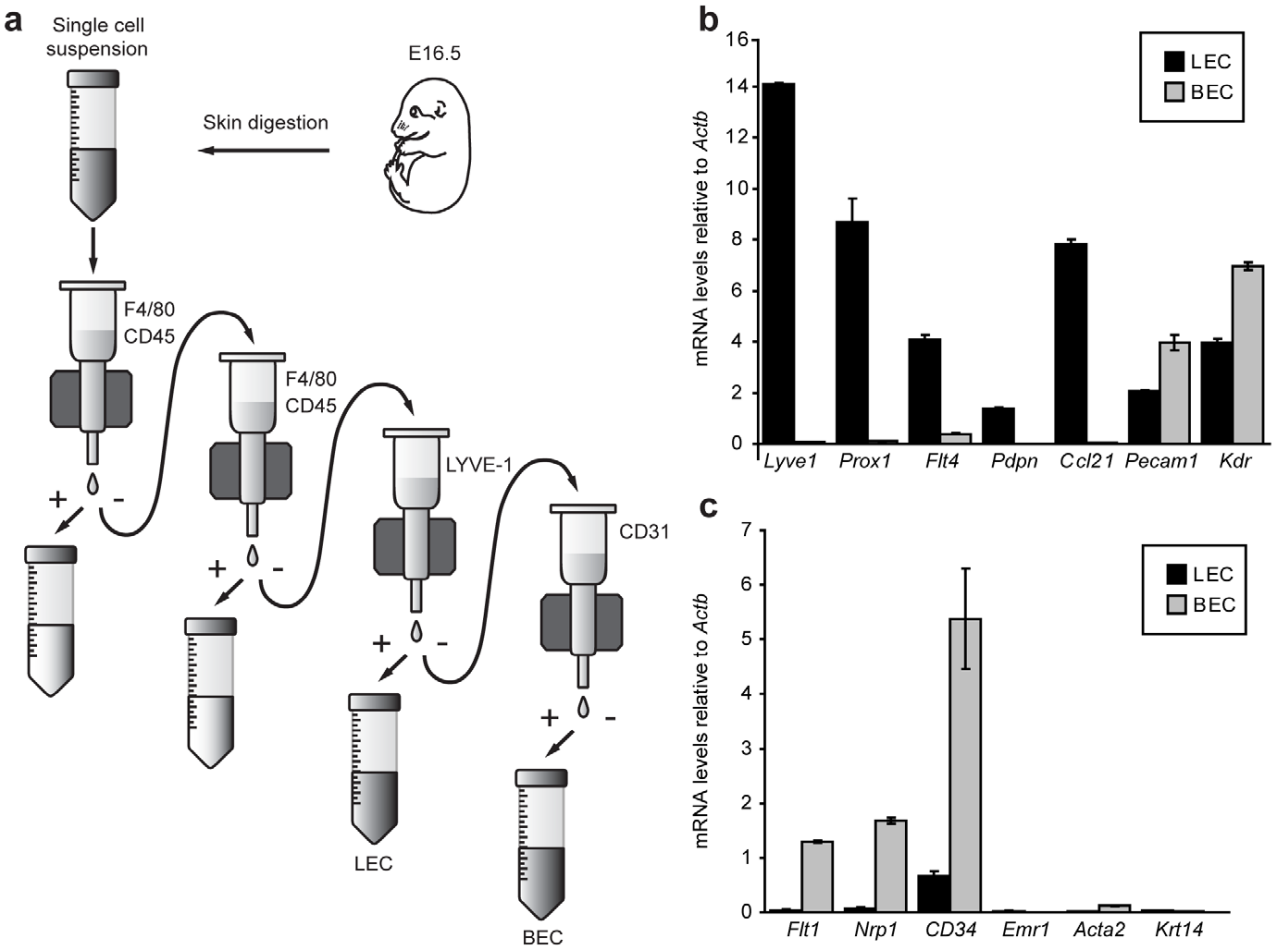
Isolation and purity of primary mouse embryonic dermal lymphatic (LEC) and blood vascular (BEC) endothelial cells. (a) Schematic representation of mouse embryonic dermal endothelial cell isolation. Skin of E16.5 embryos was removed and digested to generate a single cell suspension. Macrophages and hematopoietic cells were depleted using anti-F4/80 and anti-CD45 antibodies, in combination with anti-rat magnetic beads. LEC were captured using anti-LYVE-1 antibody and anti-rabbit magnetic beads, prior to isolation of BEC using anti-CD31 antibody and anti-rat magnetic beads. (b) Analysis of mRNA levels of established markers of LEC identity in LEC and BEC isolated from E16.5 dermis. (c) Analysis of mRNA levels of known markers of BEC (*Flt1*, *Nrp1*, *Cd34*), macrophage (*Emr1*), vascular smooth muscle (*Acta2*) and keratinocyte (*Krt14*) identity in LEC and BEC isolated from E16.5 dermis. Data were normalised to *Actb* and show mean ± s.d. of triplicate samples. Data are representative of at least three independent cell isolations, each prepared from multiple litters of embryos.

Here, we describe methodology for the isolation, short-term culture and transfection of highly pure populations of primary embryonic mouse LEC and BEC. Furthermore, we have optimised robust assays to quantify primary mouse LEC proliferation, migration and three-dimensional tube formation *in vitro*. We have utilised these techniques to investigate the roles of FGF2 and VEGF-C signalling in lymphangiogenesis *in vitro* and report that FGF2 and VEGF-C drive distinct cellular events; FGF2 potently promotes LEC proliferation while VEGF-C stimulates LEC sprouting and elongation. Our data uncover a previously unrecognised role for signal transduction via FGFR1 in primary mouse LEC and suggest that this signalling axis is likely to play a key role during lymphangiogenesis *in vivo*.

**Figure 2:**
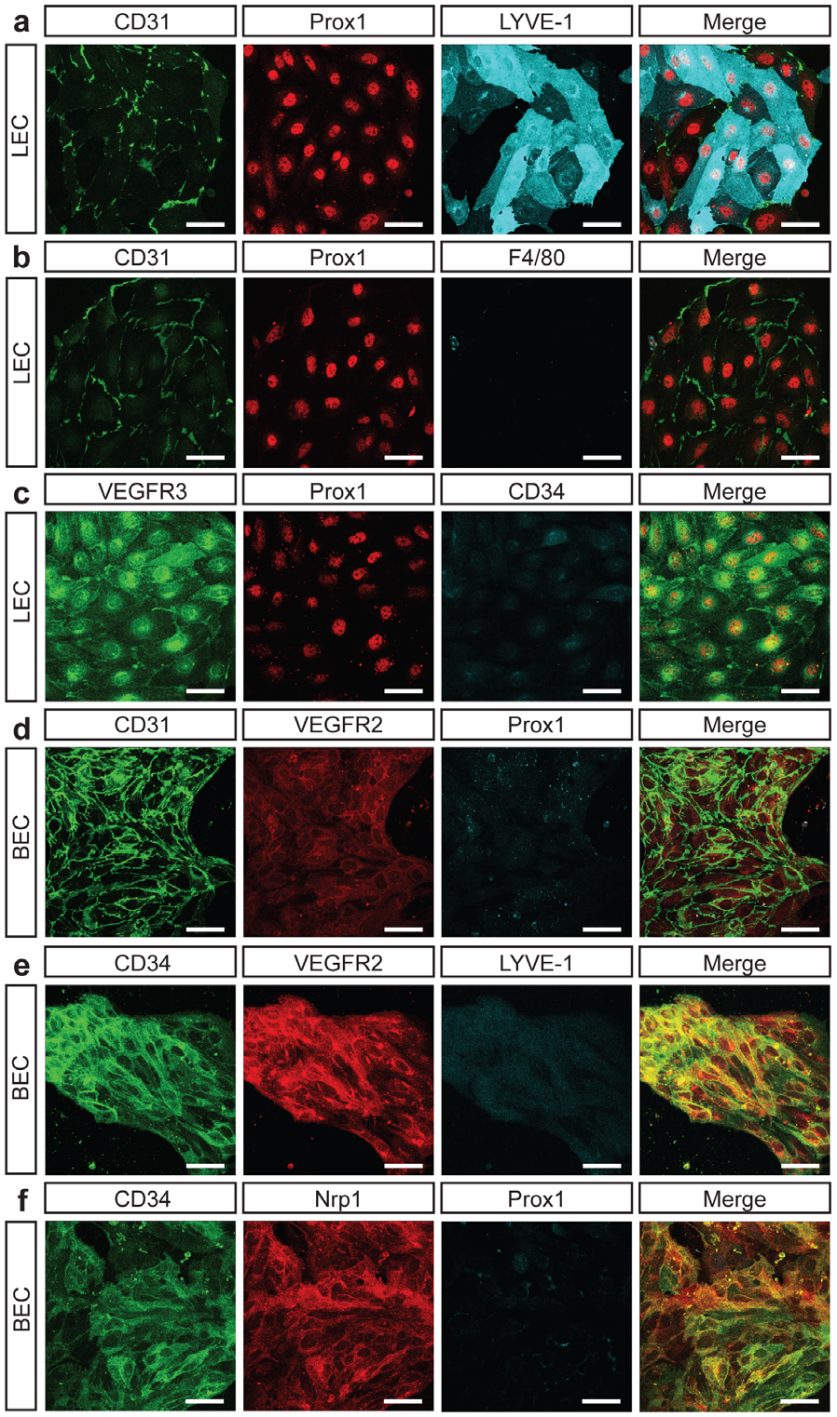
Purity of isolated embryonic dermal LEC and BEC populations assessed by immunostaining. Analysis of markers indicative of LEC and BEC identity on cells isolated from E16.5 dermis and cultured in EGM-2MV for 72 h. LEC are uniformly Prox1 and CD31 positive and depleted of hematopoietic cells. BEC are uniformly CD31, CD34, VEGFR2 and Nrp1 positive and devoid of LEC and hematopoietic cells. Scale bars represent 40 µm.

## Methods

### Animal studies and Ethics Statement

Experiments using mice were performed using C57Bl/6 mice and were approved and conducted in accordance with the SA Pathology/Central Health Network (CHN) Animal Ethics Committee and Australian National Health and Medical Research Council (NHMRC) guidelines.

**Figure 3:**
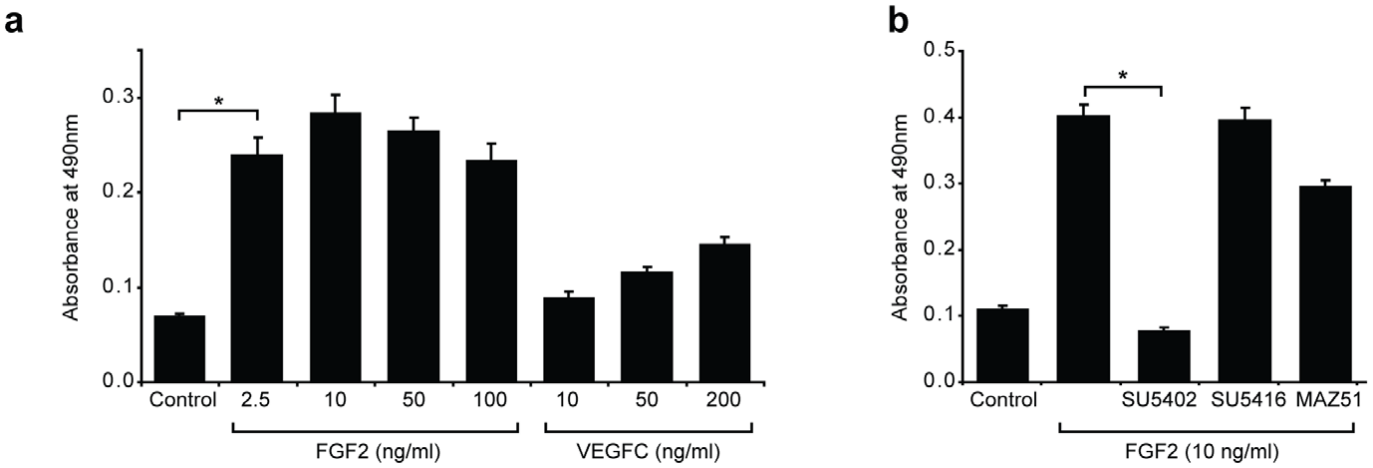
FGF2 stimulates primary mouse LEC proliferation. (a) Primary LEC were cultured in EBM-2+0.5 mg ml^−1^ Albumax (Control) or EBM-2+0.5 mg ml^−1^ Albumax containing FGF2 or VEGFC at the indicated concentrations for 48 h. LEC proliferation was measured using the CellTiter 96® AQueous One Solution Cell Proliferation Assay (Promega). Data shown represent mean ± s.e.m. and are derived from 3 independent cell isolations, each prepared from multiple litters of embryos, and 5 replicates of each treatment (n = 15). (b) FGF2 stimulated LEC proliferation is inhibited by an FGFR tyrosine kinase inhibitor but not by VEGFR inhibitors. Primary LEC were cultured in EBM-2+0.5 mg ml^−1^ Albumax (Control), or EBM-2+0.5 mg ml^−1^ Albumax and FGF2 (10 ng ml^−1^), together with the tyrosine kinase inhibitors SU5402 (10 µM, FGFR inhibitor), SU5416 (5 µM, VEGFR-2 inhibitor) or MAZ51 (5 µM, VEGFR-3 inhibitor). LEC proliferation was measured using the CellTiter 96® AQueous One Solution Cell Proliferation Assay (Promega). Data shown represent mean ± s.e.m. and are derived from 3 independent cell isolations prepared from multiple litters of embryos and 5 replicates of each treatment (n = 15). **P<0.01 ***P<0.001.

### Reagents and Antibodies

We used Dulbecco's Modified Eagle's Medium (DMEM, Sigma), Hank's Balanced Salt Solution (HBBS, Sigma), recombinant human FGF basic 146 aa (R&D Systems), recombinant human VEGF-C (R&D Systems), SU5402 (Merck), SU5416 (Sigma-Aldrich), MAZ51 (Sigma-Aldrich), rabbit anti-mouse Prox1 (AngioBio), goat anti-human Prox1 (R&D Systems), rabbit anti-mouse LYVE-1 (AngioBio), rat anti-mouse CD31 (BioLegend), rat anti-mouse CD45 (BD Pharmingen), rat anti-mouse CD34 (eBioscience), rat anti-mouse F4/80 (Invitrogen), goat anti-mouse VEGFR2 (R&D Systems), goat anti-mouse VEGFR3 (R&D Systems), rabbit anti-KDR (Upstate/Millipore), goat anti-rat Neuropilin-1 (R&D Systems), rabbit anti-Flg (FGFR1) (C-15) (Santa Cruz).

**Figure 4:**
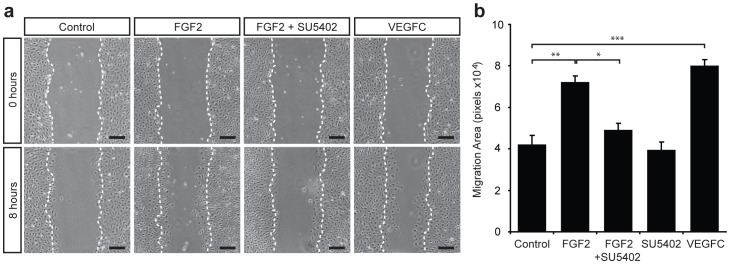
FGF2 and VEGF-C promote migration of primary mouse LEC. (a) Confluent monolayers of primary LEC were scratched and cultured in EBM-2+0.5% FBS (Control), or EBM-2+0.5% FBS containing FGF2 (10 ng ml^−1^) ± SU5402 (10 µM) or VEGF-C (200 ng ml^−1^) for 8 h. Dotted white lines mark the boundaries of the wound at 0 h. Scale bars represent 125 µm. (b) Quantification of area migrated in 8 h. Data represent mean ± s.e.m. and are derived from 3 independent cell isolations, each prepared from multiple litters of embryos, and 5 replicates of each treatment (n = 15). *P<0.05, **P<0.01, ***P<0.001.

### Preparation of skin cell suspensions

Where practicable all procedures were carried out on ice and as quickly as possible. Intact uteri were removed from 2–3 pregnant female mice and placed in ice-cold DMEM (Sigma) until dissection. Skin was removed in large pieces from 15–25 embryos and transferred to ice-cold 10 ml HHF (5% FBS, 10 mM Hepes Buffer in HBBS) until all embryos were processed. Dissected skin was rinsed twice with DHF (DMEM/20%FCS/10 mM Hepes) and replaced with 10 ml DHF containing 25 mg Collagenase Type II, 25 mg Collagenase Type IV and 10 mg Deoxyribonuclease I (all from Worthington), followed by incubation at 37°C for 30 min, pipetting with a wide-bore transfer pipette every 5 min to assist tissue dissociation. Skin cell suspensions were filtered through a 40 µm cell strainer and rinsed with 2 volumes ice-cold DHF. Filtrates were centrifuged at 300 g for 10 min. Cell pellets were resuspended in 5 ml HHF and cell counts performed. Cell suspensions from adult skin were prepared as follows: ears from 3–4 adult mice were peeled apart, excess cartilage was removed and remaining skin was cut into small pieces. Tissue digestion was carried out at 37°C for 60 min with frequent pipetting and single cell suspensions were processed as described above.

**Figure 5:**
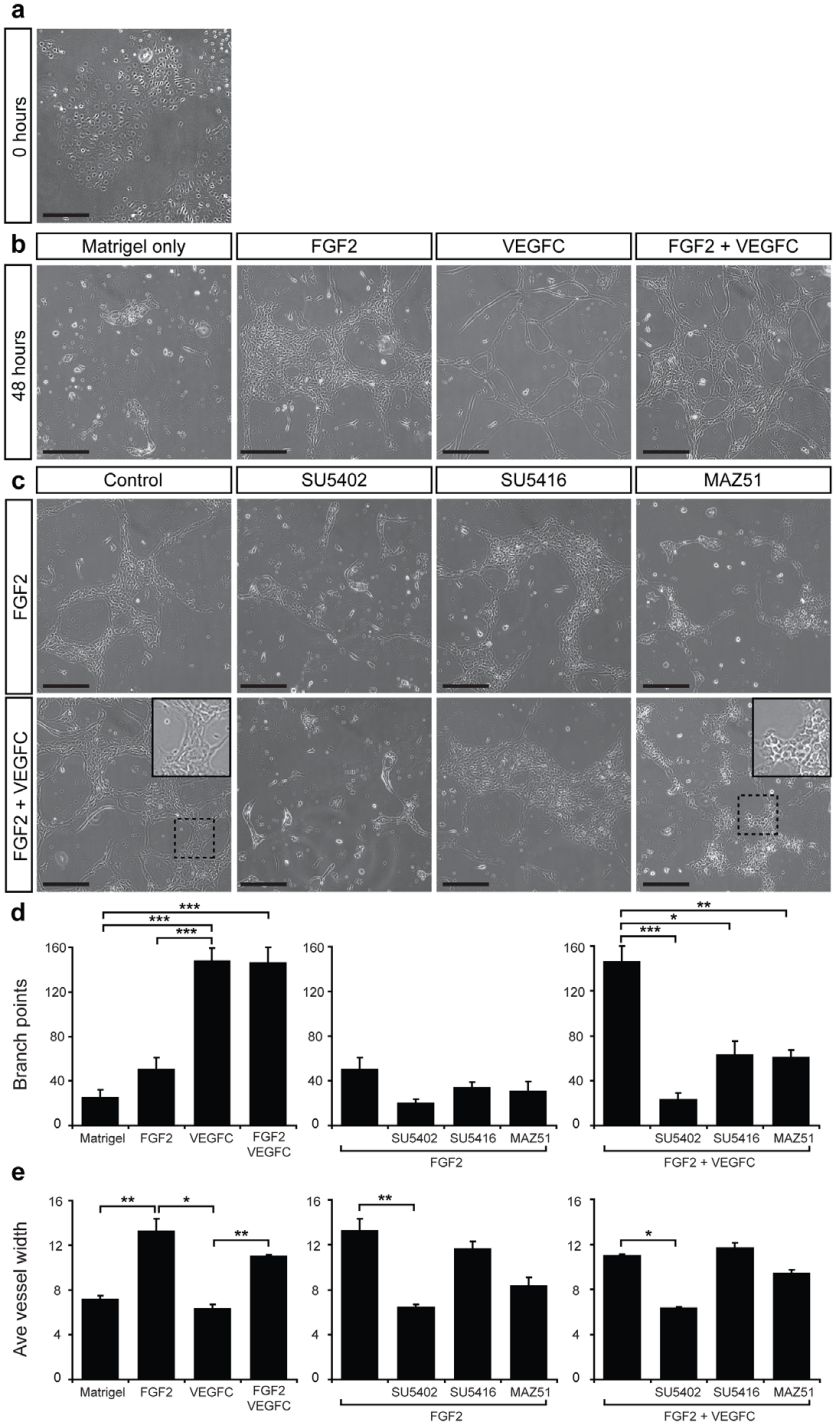
FGF2 and VEGF-C promote tube formation of primary mouse LEC. (a) Primary LEC were cultured for 24 h and imaged immediately following the addition of Matrigel. (b) Primary LEC were cultured for 24 h followed by addition of Matrigel alone or Matrigel containing FGF2 (10 ng ml^−1^), VEGF-C (200 ng ml^−1^) or a combination of FGF2 and VEGF-C. Images were captured after a further 48 hours. (c) Primary LEC were cultured for 24 h followed by addition of Matrigel containing FGF2 (10 ng ml^−1^) or a combination of FGF2 (10 ng ml^−1^) and VEGF-C (200 ng ml^−1^) and tyrosine kinase inhibitors SU5402 (10 µM, FGFR1), SU5416 (5 µM, VEGFR-2) or MAZ51 (5 µM, VEGFR-3). Three replicates of each treatment were performed and images are representative of at least three independent cell isolations. Inset panels in (c) illustrate magnified views of boxed regions. Scale bars represent 250 µm. Quantification of average vessel diameter (d) using Lymphatic Vessel Analysis Protocol (LVAP) [28] and ImageJ [29] software and branch points per well (e) using AngioTool software [30], for each treatment indicated. Data show mean ± s.e.m. and are derived from 2 independent cell isolations, each prepared from multiple litters of embryos, and 3 replicates of each treatment (n = 6). *P<0.05, **P<0.01, ***P<0.001.

### Depletion of F4/80(+)/CD45(+) haematopoietic cells

Crude dermal cell mixtures were centrifuged at 400 g for 5 min and pellets resuspended at approximately 10^8^ cells ml^−1^ in HHF together with anti-F4/80 and anti-CD45 antibodies (1∶100). Cells were mixed by gentle rotation at 4°C for 5 min, washed with 10–20 volumes MACS Buffer (PBS, 2 mM EDTA, 5% FBS), centrifuged as above and resuspended at 10^8^ cells ml^−1^ in MACS Buffer. F4/80(+)/CD45(+) cells were depleted using goat anti-rat IgG Microbeads (MiltenyiBiotec), using 200 µl beads per 10^8^ cells, according to manufacturer's instructions. F4/80(−)/CD45(−) negative cells were collected and the entire process repeated to ensure maximal depletion of contaminating haematopoietic cells.

**Figure 6:**
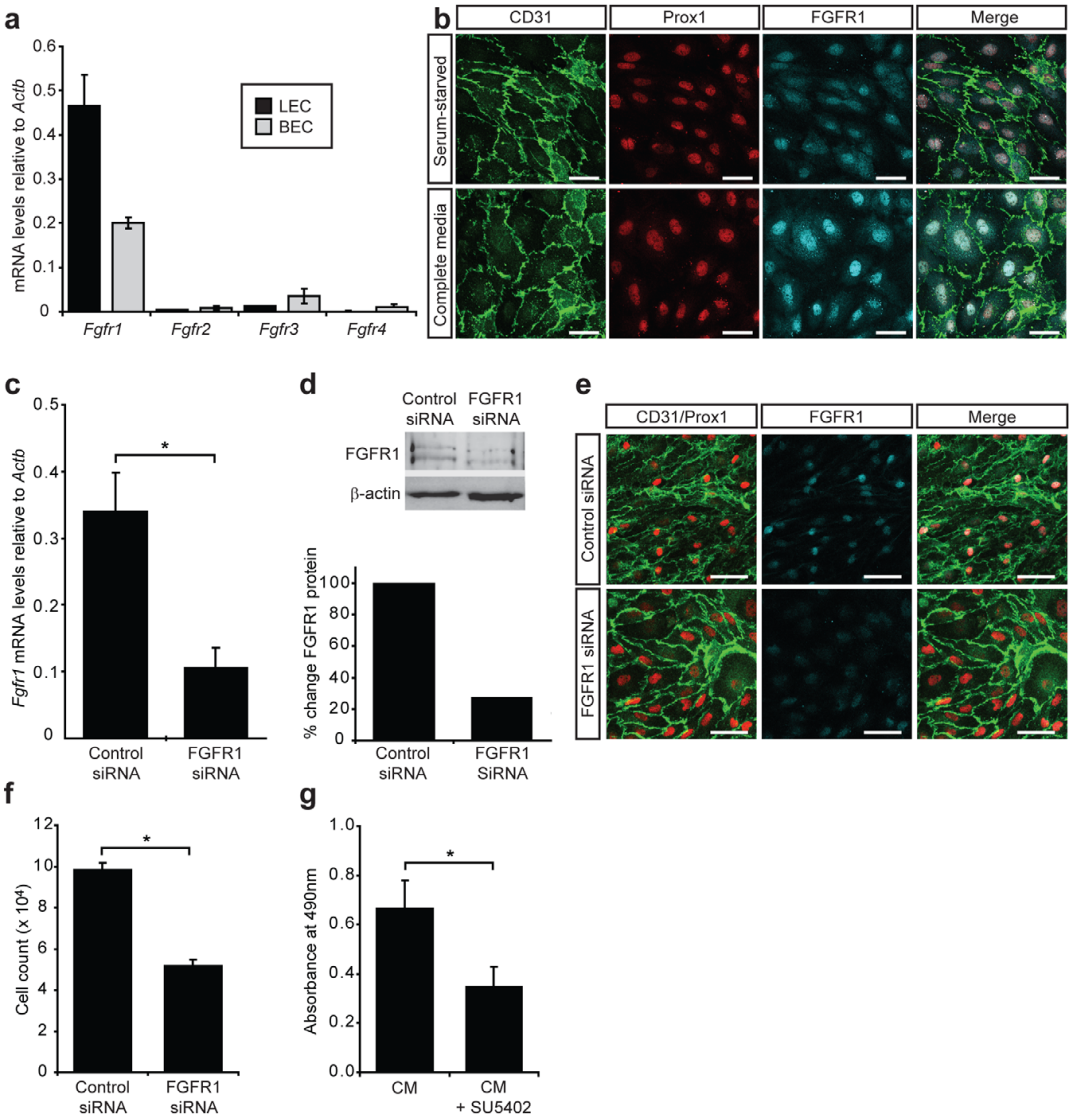
FGFR1 is important for LEC proliferation. (a) FGF receptor profile in primary embryonic mouse dermal LEC and BEC. Real-time RT-PCR analysis of *Fgfr1-4* mRNA levels in freshly isolated E16.5 LEC and BEC. Data are normalised to *Actb* and show mean ± s.d. of triplicate samples. Data are representative of at least three independent cell isolations from multiple litters of embryos. (b) FGFR1 is localized to the nucleus of LEC following stimulation with FGF2. Immunostaining of primary LEC cultured for 24 h in either EBM-2+0.5 mg ml^−1^ Albumax (serum starved) or EGM-2MV containing FGF2 (complete media). Scale bars represent 40 µm. (c) siRNA mediated knockdown of FGFR1 in primary embryonic LEC. Primary LEC were cultured for 24 h prior to transfection with control or *Fgfr1* siRNA. *Fgfr1* mRNA levels were analysed 72 h post-transfection. Data are normalised to *Actb* and represent mean ± s.e.m. Data are derived from 3 independent cell isolations, each prepared from multiple litters of embryos, and 3 transfections per isolation (n = 9). ***P<0.001. (d) FGFR1 protein levels were assessed by Western blot 72 h post-transfection and quantified relative to β-actin. (e) Immunostaining of primary LEC cultured in complete medium for 72 h after transfection with control or *Fgfr1* siRNA revealed efficient reduction in FGFR1 protein levels. Scale bars represent 100 µm. (f) FGFR1 is important for LEC proliferation. Primary LEC were cultured for 24 h prior to treatment with control or *Fgfr1* siRNA. LEC proliferation was measured by counting cells 72 h post-transfection. Data show mean ± s.e.m. Data are derived from 2 independent cell isolations, each prepared from multiple litters of embryos and multiple transfections per isolation (n = 11). ***P<0.001. (g) Primary LEC were cultured in EGM-2MV (complete media, CM) for 24 h prior to treatment with SU5402 (10 µM) for 72 h. LEC proliferation was measured using the CellTiter 96® AQueous One Solution Cell Proliferation Assay (Promega). Data shown represent mean ± s.e.m. and are derived from 3 independent cell isolations prepared from multiple litters of embryos and multiple replicates of each treatment (n = 14). *P<0.05.

### Isolation of Lyve1(+) LEC and CD31(+) BEC

F480(−)/CD45(−) cells were centrifuged at 400 g for 5 min and resuspended at approximately 10^8^ cells ml^−1^ in HHF with anti-Lyve1 antibody (1∶200). Lyve1(+) LEC were purified using goat anti-rabbit IgG Microbeads according to manufacturer's instructions and eluted cells reapplied promptly to a fresh MACS MS column to increase purity. F480(−)/CD45(−)/Lyve1(−) cells were centrifuged at 400 g for 5 min and resuspended at approximately 10^8^ cells ml^−1^ in HHF with anti-CD31 antibody (1∶100). CD31(+) BEC were purified using goat anti-rat IgG Microbeads according to manufacturer's instructions and eluted cells reapplied to a fresh MACS MS column. Isolated primary dermal LEC and BEC were either used immediately for RNA and protein isolation, or plated on fibronectin (50 µg ml^−1^, Roche) coated dishes in EBM-2 medium supplemented with EGM-2MV SingleQuots (Lonza) and grown at 37°C in 5% CO_2_.

Approximate yields:

20 embryos2–4×10^7^ total cells (approx 0.6% LEC, 2% BEC)

20 embryos1–2×10^8^ total cells (approx 1% LEC, 4% BEC)

5 embryos1–2×10^8^ total cells (approx 1% LEC, 4% BEC)

### Transfection with siRNA

LEC transfection with siRNA was performed using LipofectamineTM 2000 (Invitrogen) according to manufacturer's instructions. The sequence of the siRNA used to target *Fgfr1* (NM_010206.2, 5′-GAAGACUGCUGGAGUUAAUTT-3′) was designed and synthesized by Shanghai GenePharma Co., td. Briefly, freshly isolated LEC were seeded on fibronectin (50 µg ml^−1^, Roche) coated 24-well dishes or μ-Slides 8 well (Ibidi) at a density of 0.5–1×10^5^ cells/well, or on fibronectin coated 96-well plates at 2×10^5^ cells/well. Cells in 24-well dishes were cultured overnight and transfected with 20 pmol siRNA (33 nM final concentration). After a further 24 h, media was replaced and cells were subjected to a second transfection using 40 pmol siRNA (66 nM). Cells in 96-well plates were transfected in the same manner, using 4 and 8 pmol siRNA, respectively. Cells were harvested 48 h after the second transfection and subjected to RNA and protein analyses.

### Immunostaining

For analysis of cell purity, primary LEC and BEC isolated from E16.5 embryos were grown on fibronectin (50 µg ml^−1^, Roche) coated μ-Slides 8 well (Ibidi) for 3 days, fixed with 4% phosphate-buffered paraformaldehyde (PFA) and incubated with primary antibodies overnight at 4°C as previously described [26]. Transfected LEC were fixed 48 h after the second transfection. Alexa Fluor® -488, -555 and 647 conjugated secondary antibodies (Invitrogen) were used for visualization. Cells were mounted in Prolong Gold with DAPI (Invitrogen). Images were captured at room temperature using a Bio-Rad Radiance 2100 confocal microscope (Bio-Rad Laboratories) equipped with 3 lasers (488 nm Argon ion, 543 nm Green HeNe and 637 nm Red Diode) attached to an Olympus IX70 inverted microscope (Olympus). Adobe Photoshop CS5 version 12.0 (Adobe) was used for subsequent image processing.

### RNA Analysis

Total RNA was isolated from transfected cells using TRIzol® reagent (Invitrogen) according to the manufacturer's instructions. For investigation of mRNA expression, total RNA was reverse transcribed using Superscript III Reverse Transcriptase (Invitrogen) with a mixture of oligo dT and random hexamer primers. Primers used for real-time RT-PCR analysis are shown in Table S1. PCR was performed with RT^2^ Real-Time SYBR Green/Rox PCR master mix (SA Biosciences) and analysed on a Corbett Rotor-Gene 6000 Real-Time PCR machine. Data were normalised to the housekeeping gene *Actb* as previously described [27].

### Western Blotting

Transfected cells were either lysed using T-PER reagent including Protease Inhibitors (Thermo Scientific) or following RNA extraction using TRIzol® reagent, whereby protein was recovered from the remaining organic phase according to the manufacturer's instructions. For analysis of protein levels in transfected cells, equal volumes of protein lysate isolated from three separate transfections were pooled and electrophoresed on 8% SDS-PAGE gels prior to being transferred to PVDF (Perkin Elmer). Western blots were performed according to standard protocols using anti-Flg (C-15) (Santa Cruz) and anti-β-actin (Sigma) antibodies, followed by anti-rabbit AP (GE Healthcare) and anti-mouse Cy5 (GE Healthcare). Signal was detected using ECF Western Blot substrate (GE Healthcare) and blots were directly scanned on a Typhoon Imager (GE Healthcare). Densitometry was performed with ImageQuant TL software (GE Healthcare).

### Tube Formation assay

Freshly isolated LEC were seeded on fibronectin (50 µg ml^−1^, Roche) coated wells at a density of 10^5^ cells/0.3 ml in μ-Slides 8 well (Ibidi) or 10^4^ cells/0.01 ml in μ-Slides Angiogenesis (Ibidi) and allowed to adhere overnight. Cells were rinsed with EBM-2 and overlaid with 0.2 ml (or 0.04 ml) of Matrigel diluted 1∶1 in ice-cold EBM-2 containing growth factors and/or inhibitors at double the required final concentration. All treatments and controls were adjusted to contain the same final concentration of DMSO (0.2%). Matrigel was allowed to solidify and was further overlaid with EBM-2 containing the indicated treatments. Cells were grown for 24 or 48 h in Matrigel and images were captured using an inverted microscope (Olympus MVX10) and F-view camera (Soft Imaging System) and analysed using Cell^R^ software (Olympus Soft Imaging System). Average vessel diameter was quantified using Lymphatic Vessel Analysis Protocol (LVAP) [28] and ImageJ [29] software. The number of vessel branch points was quantified using AngioTool software [30].

### Proliferation assay

Proliferation assays were performed using the CellTiter 96® AQueous One Solution Cell Proliferation Assay reagent (Promega), as per manufacturer's instructions. All experiments were performed in 96 well plates with 4–5 replicates. Briefly, freshly isolated LEC were diluted to 1×10^5^ cells ml^−1^ in EGM-2MV (Lonza) and 0.1 ml of cells was added to each well. Plates were cultured at 37°C/5% CO_2_ for 16–18 h and then serum starved for 4 h in control media comprising EBM-2 containing 0.5 mg ml^−1^ Albumax II (Invitrogen). Control and treatment groups were added to cells and incubated for 48 h. Cell proliferation was measured either by trypsinization and cell counting using a haemocytometer, or following the addition of CellTiter 96® AQueous One Solution Cell Proliferation Assay reagent (0.02 ml) to each well and incubation for a further 4 h. Absorbance was measured at 490 nm on a FLUOstar OPTIMA microplate reader (BMG LABTECH).

### Migration scratch assay

All experiments were performed in pre-marked 96 well plates with 4–5 replicates. Freshly isolated LEC were diluted to 5×10^5^ cells ml^−1^ and 0.1 ml of cells was added to wells. Cells were cultured in EGM-2MV (Lonza) at 37°C/5% CO_2_ until confluent. Cells were then starved in EBM-2 containing 0.5% FBS (base media) overnight. A single scratch was made in each confluent cell layer using a 200 µl pipette tip and cells were washed gently in EBM-2 (Lonza). Images at time 0 (initial) were captured using an inverted phase contrast microscope (Olympus CKX41) at 4× magnification. Cells were then incubated in base media (Control) or base media containing FGF2 (10 ng ml^−1^), FGF2 (10 ng ml^−1^)+SU5402 (10 µM) or VEGF-C (200 ng ml^−1^) for 8 h. Images were captured 8 h later using an inverted phase contrast microscope (Olympus CKX41) at 4× magnification. For imaging purposes, wells were completely filled with EBM-2 to overcome distortion caused by the meniscus. Initial and final scratch areas were measured using ImageJ version 1.41 software [29] and the difference between the 0 and 8 h measurements was expressed as migration area.

### Statisitics

For proliferation and migration assays with multiple treatments, randomised complete block design with sub sampling was assumed. Data was analysed using SAS/STAT® 9.2 software and the PROC MIXED procedure. If a difference between treatments was detected at the 5% level, pair-wise comparisons of the means (with Sidak adjustment) were used to determine *P* values. Unless otherwise stated, error bars in each figure represent s.e.m. of at least three independent experiments with multiple replicates of each treatment.

## Results

### Isolation, purity and shot-term culture of primary mouse lymphatic and blood vascular endothelial cells

Primary lymphatic (LEC) and blood vascular endothelial cells (BEC) were purified from single cell suspensions of embryonic mouse skin. Cell suspensions were first depleted of hematopoietic cells including macrophages, using a magnetic bead isolation approach coupled with anti-F4/80 and anti-CD45 antibodies. Two rounds of depletion were employed to ensure complete removal of these potentially contaminating cell types. LEC were then isolated using an anti-LYVE-1 antibody and following this, BEC were selected using an anti-CD31 antibody (Figure 1a). The purity of isolated LEC and BEC was assessed immediately following cell isolation and RNA extraction, by real-time RT-PCR for a panel of genes characteristic of LEC, BEC, hematopoietic, vascular smooth muscle and epithelial identity (Figure 1b, c). These assessments revealed that genes characteristic of LEC identity including *Lyve1*, *Prox1*, *Flt4*, *Pdpn* and *Ccl21* were highly enriched in embryonic dermal LEC (Figure 1b), while genes including *Flt1*, *Nrp1* and *Cd34* were highly enriched in embryonic dermal BEC (Figure 1c). Pan-endothelial genes including *Pecam1* and *Kdr* (encoding VEGFR-2) were expressed in both LEC and BEC populations, though were higher in BEC (Figure 1b). Both primary embryonic endothelial cell populations were negative for macrophage (*Emr1*, encoding F4/80), vascular smooth muscle (*Acta2*, encoding smooth muscle actin, alpha 2) and epithelial (*Krt14*, encoding keratin 14) genes (Figure 1c), indicating a high degree of specificity in our cell isolation approach.

Purified LEC and BEC cultured on fibronectin coated dishes grew rapidly to confluence. Interestingly, the morphology of each cell type was distinct; primary dermal LEC grew in clusters and were large, oak-leaf shaped cells (Figure 2a–c). By comparison, primary dermal BEC were smaller, more spindle shaped, tended to grow in tube-like structures and grew more slowly than LEC (Figure 2d–f). These features mirror the morphology of lymphatic and blood vascular capillaries in embryonic skin; lymphatic capillaries are of a much larger calibre than their blood vascular counterparts (Figure S1a). Immunostaining for proteins characteristic of lymphatic and blood vascular capillaries in primary embryonic LEC and BEC following 3 days in culture confirmed the highly pure nature of isolated primary LEC and BEC (Figure 2, Figure S2). LEC were positive for Prox1, VEGFR-3 and CD31 and negative for macrophage (F4/80) and blood vascular markers (CD34) (Figure 2a–c). Interestingly, purified embryonic LEC, like adult mouse LEC [8], exhibited heterogeneous levels of LYVE-1 in culture (Figure 2a). BEC were positive for CD34, VEGFR-2 and Nrp1 and negative for Prox1 and LYVE-1 (Figure 2d–f). Though LEC were successfully passaged up to 4 times, cell growth and proliferation slowed with each passage. In addition, expression levels of some markers of LEC identity including *Ccl21* and thrombospondin1 (*Thbs1*) were dramatically reduced once LEC were cultured. For this reason, all of our experiments were performed with freshly isolated cells in their first passage. Intriguingly, LEC isolated from E16.5 embryos attached and proliferated more readily than LEC isolated from E18.5, perhaps reflecting elevated plasticity at this earlier developmental timepoint. Essentially the same cell isolation approach was used to purify primary LEC and BEC from adult ear skin, except that enzymatic digestion of skin cell suspensions was increased from 30 to 60 min with frequent agitation.

### FGF2 promotes primary mouse LEC proliferation

The proliferation of primary embryonic dermal LEC in culture was assessed using an established colorimetric assay for the quantification of viable cells. To investigate the effect of established pro-lymphangiogenic growth factors on the proliferation of primary embryonic dermal LEC in culture, LEC were seeded in full media (EGM-2MV) for 18 h and then starved in basal media (EBM-2 containing 0.5 mg ml^−1^ Albumax II) for 4 h. Base media was then replaced with media containing either VEGF-C or FGF2 at a range of concentrations and cell proliferation was measured 48 h later. These assays revealed that FGF2 potently stimulated the proliferation of primary embryonic dermal LEC (Figure 3a). In fact, while 2.5 ng ml^−1^ of FGF2 promoted approximately three-fold greater LEC proliferation than did base media, 200 ng ml^−1^ of VEGF-C stimulated LEC proliferation only two-fold (Figure 3a). Previous reports have ascribed a pro-lymphangiogenic role to FGF2, but suggested that this effect is mediated indirectly, via the activity of VEGF-C and VEGF-D [31], [32]. We assessed whether this was the case in primary embryonic dermal LEC by treating LEC with FGF2 in combination with small molecule tyrosine kinase inhibitors of FGFR (SU5402) [33], VEGFR-2 (SU5416) [34] and VEGFR-3 (MAZ51) [35]. The addition of SU5402 to LEC media in the presence of FGF2 completely abrogated FGF2 stimulated proliferation (Figure 3b). In contrast, small molecule tyrosine kinase inhibitors of VEGFR-2 (SU5416) and VEGFR-3 (MAZ51) that reduced VEGF-C mediated LEC proliferation (Figure S3), did not significantly inhibit FGF2-mediated LEC proliferation (Figure 3b). These data demonstrate that FGF2 promotes the proliferation of primary LEC directly via FGF receptors and independently of VEGF receptors.

### FGF2 and VEGF-C promote the migration of primary mouse LEC

We next established an assay to quantify the migration of primary LEC in response to lymphangiogenic stimuli. We first assessed the ability of LEC seeded in Boyden chambers to migrate through a filter towards VEGF-C, but found that only a very low proportion of cells were capable of chemotactic migration through the filter, perhaps due to detrimental effects of trypsinization prior to cell seeding. We next established a “scratch” assay to quantify cell migration. For this assay, freshly isolated LEC were allowed to grow to confluence and were then washed and starved for 16 h. Scratches across LEC monolayers were made and LEC migration in response to designated treatments was measured following 8 h. A dose response assay revealed that maximal LEC migration was achieved at a dose of 10 ng ml^−1^ FGF2 (Figure S4) and that this dose of FGF2 promoted the migration of primary embryonic dermal LEC to a similar extent as 200 ng ml^−1^ VEGF-C (Figure 4). Moreover, FGF2 stimulated LEC migration was inhibited by the small molecule FGFR inhibitor SU5402 (10 µM), illustrating that FGF2 promoted LEC migration directly, via cell autonomous signalling through FGF receptors.

### FGF2 cooperates with VEGF-C to promote tube formation

We next sought to establish a three-dimensional assay able to reproduce features of lymphatic vessel growth *in vivo*, and to this end, developed a protocol to promote the assembly of primary embryonic dermal LEC into tubes. We initially attempted to promote LEC tube formation in an analogous fashion to methods used to stimulate the formation of human LEC tubes, by plating LEC onto Matrigel [9]. However, primary embryonic mouse dermal LEC did not form tubes in this setting. To promote tube formation, we first established semi-confluent (50–70%) cultures of primary dermal LEC (Figure 5a) and then overlaid LEC monolayers with Matrigel containing selected growth factors and/or small molecule inhibitors. Tube formation was measured 24 and 48 h later. Primary embryonic mouse dermal LEC rapidly organised into aggregates following the addition of Matrigel (Figure 5b). However, in the presence of FGF2 (10 ng ml^−1^), LEC dynamically organised into a network of tubes and exhibited features characteristic of lymphatic vessel growth including proliferation, sprouting, migration and anastomosis (Figure 5b). The effects of growth factor treatment on tube formation were quantified by measuring average vessel diameter and the number of vessel branchpoints per field. Cell proliferation was a striking feature of FGF2-promoted tube formation, reflected in the increased diameter of LEC tubes following treatment with FGF2 compared to Matrigel alone (Figure 5b, d). The addition of VEGF-C to Matrigel also enhanced LEC tube formation, but the morphology of tubes formed in response to VEGF-C treatment was strikingly different to that promoted by FGF2. VEGF-C potently promoted LEC sprouting and elongation, rather than proliferation (Figure 5b); VEGF-C induced tubes contained substantially more branchpoints than FGF2-induced tubes, (Figure 5e), while vessel diameter was significantly lower (Figure 5d). In combination, FGF2 and VEGF-C promoted proliferation and sprouting, resulting in the development of large, interconnected lymphatic vascular tubes (Figure 5b, d, e). Tube formation promoted by FGF2 was inhibited by the small molecule FGFR inhibitor SU5402, but not by the VEGFR-2 inhibitor SU5416 (Figure 5c, d, e). Intriguingly, FGF2 promoted tube formation was significantly affected by treatment with the VEGFR-3 inhibitor MAZ51 (Figure 5 c, d). In particular, vessel diameter and the morphology of cells were altered; LEC assumed a more cuboidal, less elongated morphology (Figure 5c) in response to MAZ51 treatment.

### FGFR1 is the predominant FGF receptor in primary dermal mouse LEC and is crucial for LEC proliferation

In order to investigate which FGF receptors were responsible for transducing FGF2 mediated pro-lymphangiogenic signals, we first quantified the mRNA expression level of each of the four FGF receptors in primary dermal LEC and BEC immediately following cell isolation from the skin. Real-time RT-PCR of embryonic and adult LEC revealed that *Fgfr1* was by far the predominant FGF receptor in both embryonic (Figure 6a) and adult (Figure S5) LEC and BEC. Moreover, *Fgfr1* levels were more than two-fold elevated in LEC compared to BEC, providing a possible explanation for the sensitivity of LEC to low doses of FGF2. In comparison, *Fgfr3*, previously suggested to have a pro-lymphangiogenic role [36], was expressed at dramatically lower levels than *Fgfr1* in primary embryonic (Figure 6a) and adult (Figure S4) LEC. Investigation of FGFR1 protein levels in primary embryonic LEC revealed that FGFR1 was localised primarily to the nucleus of LEC grown in complete media (Figure 6b). Nuclear FGFR1 has previously been associated with cell proliferation [37]. In order to establish whether this was also the case in primary embryonic LEC, FGFR1 localisation was compared between cells grown in complete media and cells that were serum starved. Substantially more nuclear FGFR1 was observed in LEC grown in complete media containing FGF2, corresponding to elevated LEC proliferation under these conditions (Figure 6b).

We next set out to investigate the requirement for FGFR1 in FGF2-mediated proliferation. Due to the reported inhibition of FGFR3 by SU5402 [38], we sought to specifically ablate FGFR1 activity using siRNA to knockdown FGFR1 levels in primary LEC. Transfection of primary embryonic dermal LEC with *Fgfr1*, but not control siRNA, reduced *Fgfr1* mRNA levels by ∼70% (Figure 6c) within 48 h of transfection. *Fgfr3* mRNA levels were not affected following transfection of LEC with *Fgfr1* siRNA (Figure S6), demonstrating specificity of *Fgfr1* siRNA for its intended target. A corresponding decrease in FGFR1 protein levels by ∼70% was also observed in *Fgfr1* siRNA treated cells (Figure 6d, e). Importantly, transfection of primary embryonic LEC with *Fgfr1* siRNA inhibited LEC proliferation in full media containing FGF2 (Figure 6f) to a similar extent as treatment with SU5402 (Figure 6g). These data reveal that FGFR1 plays a key role in LEC proliferation and suggest that signal transduction via FGFR1 is likely to be important for lymphangiogenesis *in vivo*.

## Discussion

Though primary mouse endothelial cells have traditionally proven difficult to culture, we have developed robust methodology for the measurement of primary embryonic mouse LEC proliferation, migration and three-dimensional tube forming ability in response to pro-lymphangiogenic stimuli *in vitro*. Moreover, we have utilised these techniques to demonstrate that FGF2 potently promotes LEC proliferation, migration and tube formation and that these activities rely on signal transduction via FGFR1. Our ability to dissect FGF signalling in a cell autonomous manner has revealed that FGF2 promotes LEC proliferation independently of VEGF receptors and moreover, that FGF2 and VEGF-C play distinct roles in lymphatic vascular morphogenesis. The assays we have developed fill a longstanding gap in the field and provide the opportunity to rapidly and precisely determine gene function and delineate signalling pathway activity in primary embryonic LEC isolated from both wild-type and genetically modified mice *ex vivo*.

While long recognised as a pro-lymphangiogenic factor, the mechanisms by which FGF2 acts on LEC have, to date, been poorly understood. In contrast to previous work suggesting that FGF2 promotes lymphangiogenesis indirectly, via increasing VEGF-C and VEGF-D production and VEGFR-3 signalling [31], [32], our assays revealed that FGF2 potently promotes the proliferation of primary embryonic mouse LEC directly, via FGFR1. Though our studies focussed on embryonic mouse LEC, previous work has shown that FGF2 stimulates the proliferation and migration of postnatal bovine, human and rat LEC *in vitro* [4], [15], [36] and promotes lymphangiogenesis when ectopically introduced to adult mouse tissues *in vivo* [31], [32]. On the basis of these data, we predict that primary adult mouse LEC would also be responsive to FGF2. Whether FGF2 primarily activates Ras/MAPK, Plcγ/Ca^2+^ or PI3K/Akt pathways (the 3 major pathways activated by FGF signalling [39]) in primary embryonic mouse LEC downstream of FGFR1 remains to be established.

Our assays revealed distinct effects of FGF2 and VEGF-C in cellular functions important for lymphangiogenesis. While FGF2 potently promoted LEC proliferation, VEGF-C primarily promoted cell sprouting and together, these growth factors cooperatively induced the formation of lymphatic vascular tubes. The effect of the small molecule inhibitor of VEGFR-3, MAZ51, on the morphology of primary LEC in a three dimensional environment confirmed that signalling via VEGFR-3 is particularly important for LEC sprouting and elongation. Taken together, these data demonstrate that the assays we have developed are useful tools with which to dissect pro-lymphangiogenic signalling pathways in primary embryonic mouse LEC. Further elucidating the downstream effectors of FGFR1 and VEGFR-3 signalling pathways that are responsible for LEC proliferation versus sprouting will provide important new insights into how morphogenetic events important for lymphangiogenesis are regulated. Moreover, our data pave the way for future experiments to characterise the interplay between FGF and VEGF signalling pathways in primary lymphatic endothelial cells.

Our discovery that FGFR1 is the predominant FGF receptor in primary, non-cultured embryonic mouse LEC and that FGFR1 receptor levels are substantially higher in LEC than BEC, provides an explanation for the potent stimulation of LEC proliferation by FGF2. Furthermore, our finding that embryonic LEC proliferation is dependent on FGFR1 provides an explanation for the observation that knockdown of FGFR3, identified as a gene induced in response to ectopic Prox1 expression in BEC, does not completely block FGF2 stimulated proliferation of human LEC [36]. Further evidence of a predominantly pro-proliferative role for FGFR1 in LEC was provided by our finding that in response to the addition of growth media containing FGF2, FGFR1 accumulated in the nucleus of LEC. Nuclear translocation of FGFR1 has previously been shown to correlate with transcriptional activation and cell proliferation [37], [40]. Defining the proteins that interact with nuclear FGFR1, together with the transcriptional targets of FGFR1 in primary LEC will no doubt shed further light on the mechanisms by which FGFR1 activation promotes LEC proliferation. The early embryonic lethality of *Fgfr1^−/−^* mice [41], [42] has, to date, precluded analysis of the role of this receptor in blood and lymphatic vascular development, though the recent generation of *Fgfr1^flox/flox^* mice [43], together with blood and lymphatic vascular specific Cre lines, now enable this question to be addressed. Our data provide compelling evidence in support of a crucial role for FGF signalling in lymphangiogenesis and pave the way for further analysis of FGFR1 function in developmental and disease stimulated lymphangiogenesis *in vivo*.

## Supporting Information

Figure S1 **Vascular morphology and marker expression.** (a) Whole mount immunostaining of E14.5 skin illustrating that the calibre of lymphatic capillaries (Prox1-positive, Nrp2-positive, CD31-positive) is substantially larger than that of blood vascular capillaries (Prox1-negative, Nrp2-negative, CD31-positive). (b) LYVE-1 levels are heterogeneous on lymphatic capillaries, while Nrp2 levels appear uniform. Scale bars represent 120 µm.
(TIF)

Figure S2 **Purity of isolated primary embryonic mouse LEC.** Immunostaining of purified primary embryonic LEC cultured in EGM-2MV demonstrating that the majority of DAPI-positive nuclei are positive for the lymphatic endothelial cell marker Prox1.
(TIF)

Figure S3 **VEGF-C stimulated proliferation of primary mouse LEC is inhibited by small molecule inhibitors of VEGFR-2 and VEGFR-3.** Primary LEC were cultured in EBM+0.5 mg ml^−1^ Albumax (Control) or EBM+0.5 mg ml^−1^ Albumax containing VEGF-C (200 ng ml^−1^) and the small molecule tyrosine kinase inhibitors SU5416 (5 µM, VEGFR-2) or MAZ51 (5 µM, VEGFR-3) for 48 h. LEC proliferation was measured using the CellTiter 96® AQueous One Solution Cell Proliferation Assay (Promega). Data shown represent mean ± s.e.m. and are derived from 3 independent cell isolations, each prepared from multiple litters of embryos and 4 replicates of each treatment (n = 12). ** *P*<0.01, ***P<0.001.
(TIF)

Figure S4 **FGF2 promotes migration of primary mouse LEC in a dose dependent manner.** Confluent monolayers of primary LEC were scratched and cultured in EBM+0.5% FBS containing FGF2 at the indicated concentrations. The area of LEC migration was quantified after 8 h. Data represent mean ± s.e.m. of three independent scratches using one cell isolation prepared from multiple litters of embryos (n = 3).
(TIF)

Figure S5 **FGF receptor profile in primary LEC and BEC isolated from adult mouse skin.** (a) Real-time RT-PCR analysis of *Fgfr1-4* mRNA levels in LEC and BEC freshly isolated from adult ear skin. Data are normalised to *Actb* and show mean ± s.d. of triplicate samples from one experiment. Data are representative of three independent cell isolations using ears pooled from 3–4 mice.
(TIF)

Figure S6 **siRNA mediated knockdown of FGFR1 in primary embryonic LEC does not affect** ***Fgfr3*** **levels.** Primary LEC were cultured for 24 h prior to transfection with control or *Fgfr1* siRNA. *Fgfr3* mRNA levels were analysed 72 h post-transfection. Data are normalised to *Actb* and show mean ± s.e.m. of three independent transfections (n = 3). Data are representative of 3 independent cell isolations from multiple litters of embryos.
(TIF)

Table S1 **Primers used for real-time RT-PCR analysis.**
(DOC)
