# Supplementary figures and images for: In Vitro Assays Using Primary Embryonic Mouse Lymphatic Endothelial Cells Uncover Key Roles for FGFR1 Signalling in Lymphangiogenesis
Source: PLoS One. 2012 Jul 6;7(7):e40497. doi: 10.1371/journal.pone.0040497 (PMC3391274; doi:10.1371/journal.pone.0040497)

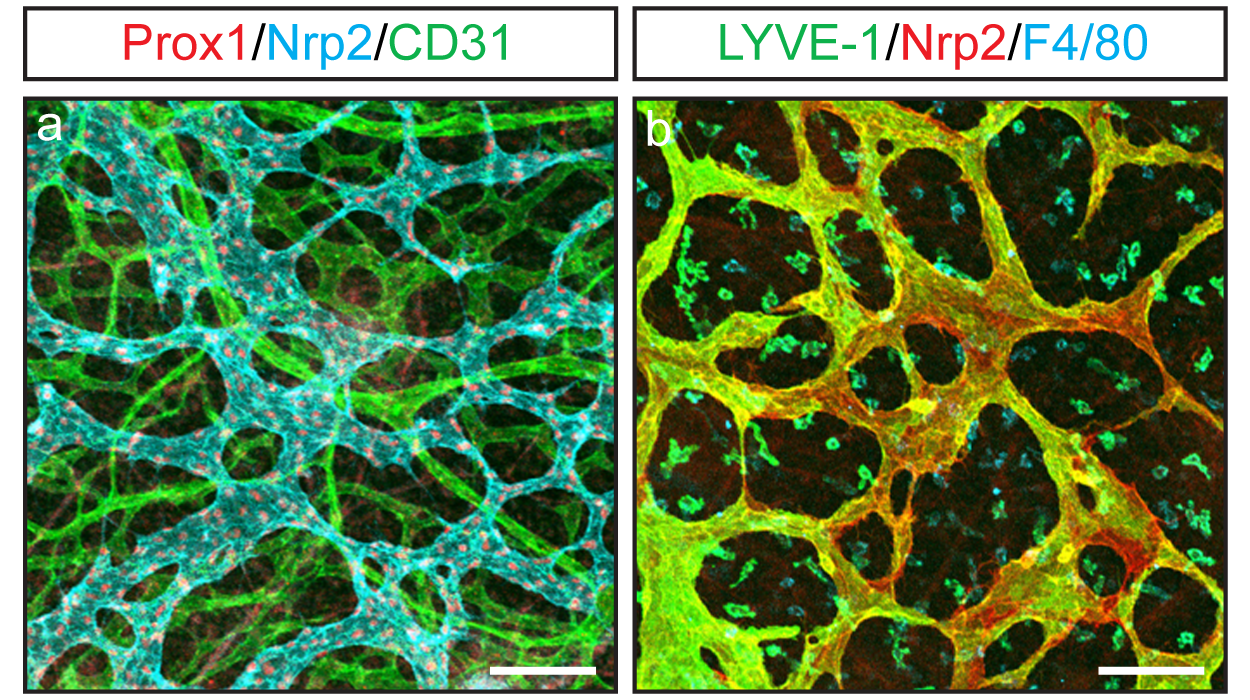

Supplement: Figure S1 — Vascular morphology and marker expression. (a) Whole mount immunostaining of E14.5 skin illustrating that the calibre of lymphatic capillaries (Prox1-positive, Nrp2-positive, CD31-positive) is substantially larger than that of blood vascular capillaries (Prox1-negative, Nrp2-negative, CD31-positive). (b) LYVE-1 levels are heterogeneous on lymphatic capillaries, while Nrp2 levels appear uniform. Scale bars represent 120 µm. (TIF) [file pone.0040497.s001.tif]

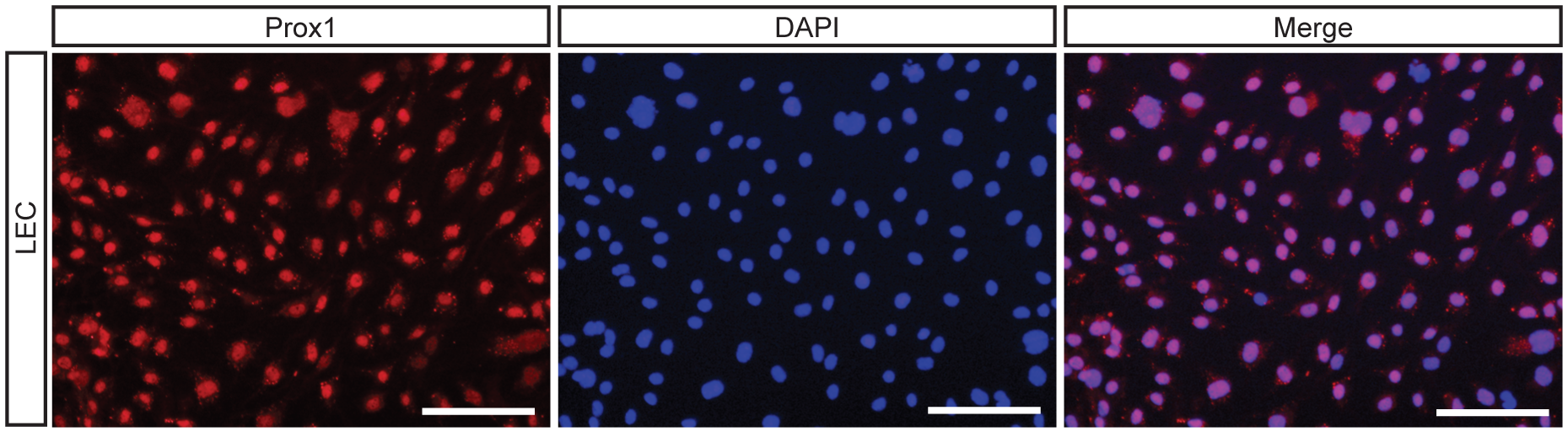

Supplement: Figure S2 — Purity of isolated primary embryonic mouse LEC. Immunostaining of purified primary embryonic LEC cultured in EGM-2MV demonstrating that the majority of DAPI-positive nuclei are positive for the lymphatic endothelial cell marker Prox1. (TIF) [file pone.0040497.s002.tif]

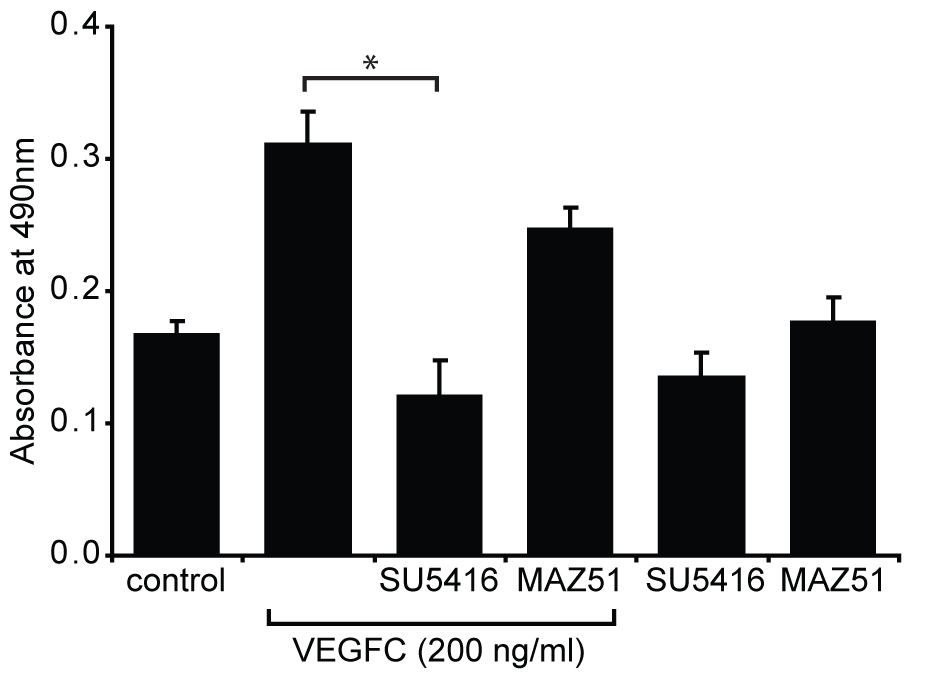

Supplement: Figure S3 — VEGF-C stimulated proliferation of primary mouse LEC is inhibited by small molecule inhibitors of VEGFR-2 and VEGFR-3. Primary LEC were cultured in EBM+0.5 mg ml−1 Albumax (Control) or EBM+0.5 mg ml−1 Albumax containing VEGF-C (200 ng ml−1) and the small molecule tyrosine kinase inhibitors SU5416 (5 µM, VEGFR-2) or MAZ51 (5 µM, VEGFR-3) for 48 h. LEC proliferation was measured using the CellTiter 96® AQueous One Solution Cell Proliferation Assay (Promega). Data shown represent mean ± s.e.m. and are derived from 3 independent cell isolations, each prepared from multiple litters of embryos and 4 replicates of each treatment (n = 12). ** P<0.01, ***P<0.001. (TIF) [file pone.0040497.s003.tif]

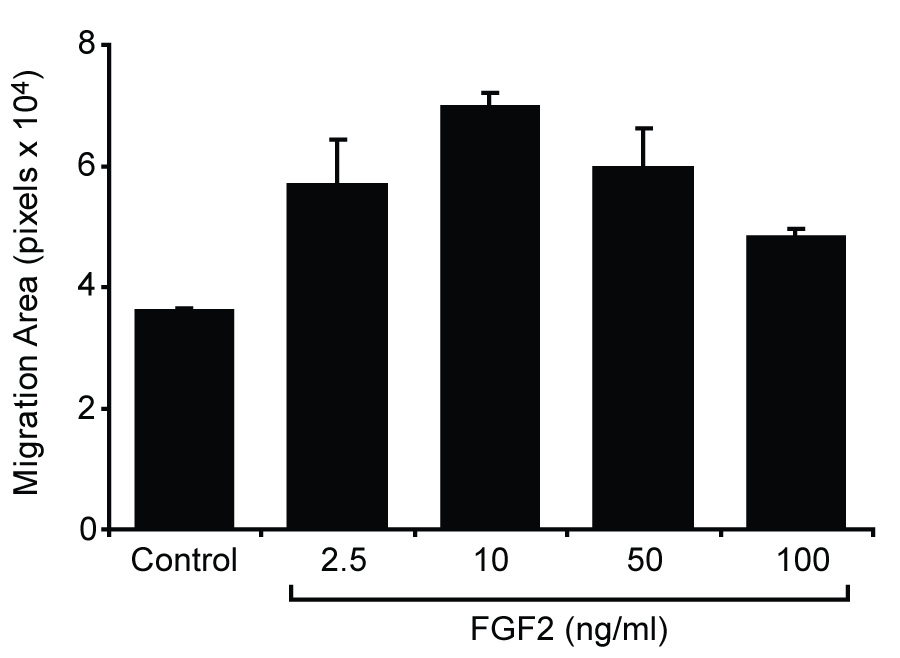

Supplement: Figure S4 — FGF2 promotes migration of primary mouse LEC in a dose dependent manner. Confluent monolayers of primary LEC were scratched and cultured in EBM+0.5% FBS containing FGF2 at the indicated concentrations. The area of LEC migration was quantified after 8 h. Data represent mean ± s.e.m. of three independent scratches using one cell isolation prepared from multiple litters of embryos (n = 3). (TIF) [file pone.0040497.s004.tif]

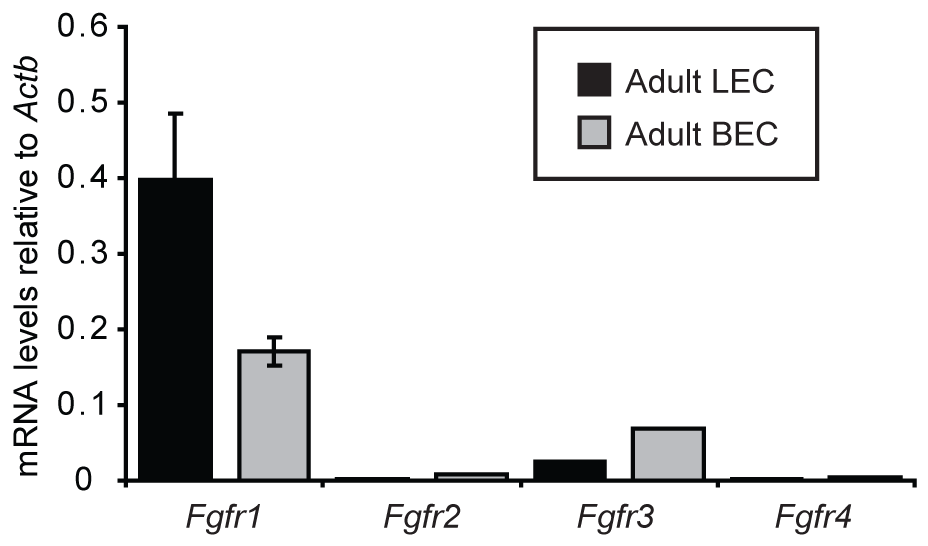

Supplement: Figure S5 — FGF receptor profile in primary LEC and BEC isolated from adult mouse skin. (a) Real-time RT-PCR analysis of Fgfr1-4 mRNA levels in LEC and BEC freshly isolated from adult ear skin. Data are normalised to Actb and show mean ± s.d. of triplicate samples from one experiment. Data are representative of three independent cell isolations using ears pooled from 3–4 mice. (TIF) [file pone.0040497.s005.tif]

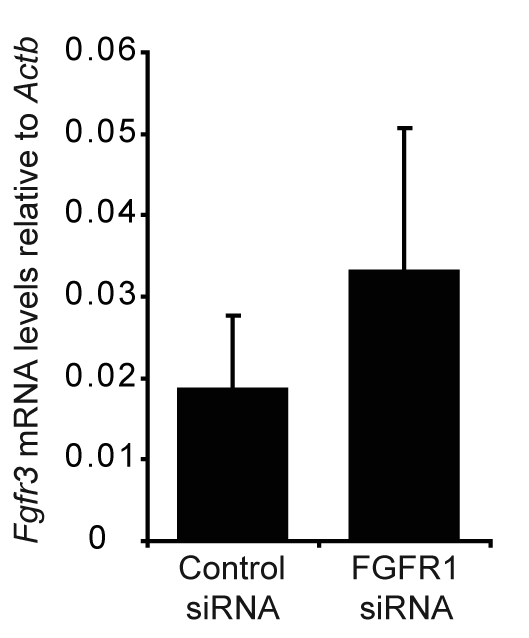

Supplement: Figure S6 — siRNA mediated knockdown of FGFR1 in primary embryonic LEC does not affect Fgfr3 levels. Primary LEC were cultured for 24 h prior to transfection with control or Fgfr1 siRNA. Fgfr3 mRNA levels were analysed 72 h post-transfection. Data are normalised to Actb and show mean ± s.e.m. of three independent transfections (n = 3). Data are representative of 3 independent cell isolations from multiple litters of embryos. (TIF) [file pone.0040497.s006.tif]
