# Supplementary material for: In Vitro Assays Using Primary Embryonic Mouse Lymphatic Endothelial Cells Uncover Key Roles for FGFR1 Signalling in Lymphangiogenesis
Source: PLoS One. 2012 Jul 6;7(7):e40497. doi: 10.1371/journal.pone.0040497 (PMC3391274; doi:10.1371/journal.pone.0040497)
Supplement: Table S1 — Primers used for real-time RT-PCR analysis. (DOC) [file pone.0040497.s007.doc]

| Gene | Forward Primer | Reverse Primer |
| --- | --- | --- |
| *Acta2* | 5’-GCATCCACGAAACCACCTAT-3’ | 5’-TGGAAGGTAGACAGCGAAGC-3’ |
| *Actb* | 5’-GATCATTGCTCCTCCTGAGC-3’ | 5’-GTCATAGTCCGCCTAGAAGCAT-3’ |
| *Ccl21* | 5’-CCACAATCATGGCTCAGATG-3’ | 5’-CTTCCTCAGGGTTTGCACAT-3’ |
| *Cd34* | 5’-TCCCCATCAGTTCCTACCAA-3’ | 5’-CAGTTGGGGAAGTCTGTGGT-3’ |
| *Emr1* | 5’-CCATTGCCCAGATTTTCATC-3’ | 5’-GGTCAGTCTTCCTGGTGAGG-3’ |
| *Fgfr1* | 5’-GGCCTCTACGCTTGCGTGACC-3’ | 5’-CCTACGGTTTGGTTTGGTGTTGTCC-3’ |
| *Fgfr2* | 5’-ACCAGGGATTGGCACTGTGACCA-3’ | 5’-ACCACGTACGCTTCTGGTTGGGA-3’ |
| *Fgfr3* | 5’-GACGGCACGCCCTACGTCAC-3’ | 5’-GGACGCCTGCGTACACGCTG-3’ |
| *Fgfr4* | 5’-GGCCCGACCAAACCAGCACC | 5’-CGGGCACGGAGGAATTCCCG-3’ |
| *Flt1* | 5’-AGCACCTTGACCTTGGACAC-3’ | 5’-CAGGGGATGATGAGCTGTCT-3’ |
| *Flt4* | 5’-CTGGCCAGAGGCACTAAGAC-3’ | 5’-CAGGGTGTCCTCTGGGAATA-3’ |
| *Krt14* | 5’-CGGCAAGAGTGAGATTTCTG-3’ | 5’-TCCAGCAGGATTTTGTACTC-3’ |
| *Lyve1* | 5’-TGGTGTTACTCCTCGCCTCT-3’ | 5’-TTCTGCGCTGACTCTACCTG-3’ |
| *Nrp1* | 5’-AAACCTTGGTGGAATTGCTG-3’ | 5’-TGGCTTCCTGGAGATGTTCT-3’ |
| *Pdpn* | 5’-ATGGCTTGCCAGTAGTCACC-3’ | 5’-TCCTCCACAGGAAGAGGATG-3’ |
| *Pecam1* | 5’-AACAGAAACCCGTGGAGATG-3’ | 5’-GTCTCTGTGGCTCTCGTTCC-3’ |
| *Prox1* | 5’-CTGGGCCAATTATCACCAGT-3’ | 5’-GCCATCTTCAAAAGCTCGTC-3’ |
